# 21-Gene Recurrence Score and Adjuvant Chemotherapy Decision for Breast Cancer Patients with Positive Lymph Nodes

**DOI:** 10.1038/s41598-019-49644-6

**Published:** 2019-09-11

**Authors:** Yiwei Tong, Jiayi Wu, Ou Huang, Jianrong He, Li Zhu, Weiguo Chen, Yafen Li, Xiaosong Chen, Kunwei Shen

**Affiliations:** Comprehensive Breast Health Center, Ruijin Hospital, Shanghai Jiao Tong University School of Medicine, Shanghai, China

**Keywords:** Breast cancer, Chemotherapy

## Abstract

The 21-gene recurrence score (RS) assay is prognostic and predictive for hormone receptor (HR)+/HER2-/node- breast cancer (BC) patients. However, its clinical value in node + patients hasn’t been elucidated. HR+/HER2-/pN1 patients operated in Comprehensive Breast Health Center, Shanghai Ruijin Hospital from January 2014 to December 2018, with available RS results were retrospectively included. Clinico-pathological characteristics were compared. Adjuvant chemotherapy recommendations pre-/post- RS assay and actual usage were analyzed. A total of 303 patients were included, with 59, 178, 66 RS < 18, 18–30 and ≥ 31. Age (*P* < 0.001), comorbidity (*P* = 0.013), and RS category (*P* < 0.001) were independently associated with chemotherapy recommendation. Compared with low RS patients, those with intermediate (OR 6.58, 95% CI 2.37–18.31, *P* < 0.001) or high (OR 54.14, 95% CI 3.77–776.54, *P* = 0.003) RS were more likely to be recommended with chemotherapy. RS independently influence chemotherapy decision in postmenopausal population as well. Chemotherapy recommendation changed for 9.57% patients after RS assay. Patient adherence rate to chemotherapy recommendation was 94.72% (287/303). The 21-gene RS independently influenced chemotherapy recommendation in pN1 BC patients, which could provide additional information to guide chemotherapy decision with relatively good treatment adherence rate.

## Introduction

Breast cancer (BC) is the most common malignant tumor in women worldwide. According to the latest global epidemiological cancer survey, an estimated 2.1 million new BC cases would be diagnosed in 2018, representing 25% of all cancer cases among women. BC is estimated to be responsible for 626,700 deaths, accounting for 6.6% of all cancer deaths^1^. As an essential part of systemic treatment, standard adjuvant chemotherapy reduces one third breast cancer mortality compared with no chemotherapy^2^. Patients with high absolute risk of disease recurrence and death gain most absolute benefit from chemotherapy, independent of classical clinico-pathological characteristics including age, hormone receptor (HR) status or node involvement^2–4^.

21-gene recurrence score (RS) is the most frequently applied multigene assay in clinical practice to provide individualized information other than routine clinico-pathological features, which can predict chemotherapy benefit and guide adjuvant treatment decision in HR-positive, human epidermal growth factor receptor 2 (HER2)-negative, and node-negative BC patients. The assay is designed to measure the expression of 21 genes including 16 cancer-related genes and 5 endogenous references in formalin-fixed paraffin-embedded (FFPE) breast tumors using quantitative reverse transcriptase polymerase chain reaction (qRT-PCR) methods^5,6^. According to the results of the prospective TAILORx trial^5,7^, the 2018 NCCN Clinical Practice Guidelines in Oncology for Breast Cancer suggest to spare selective low risk patients from adjuvant chemotherapy^8^. Meanwhile, based on the NSABP B-20 trial, chemotherapy is still recommended for high risk patients, since a 27.6% absolute decrease of 10-year distant recurrence rate was reported in high risk N0 patients receiving chemotherapy^9^.

While the current guidelines suggest the routine use of 21-gene RS testing in node-negative patients, results from several clinical trials have extended its application in patients with 1–3 histologically proven involved axillary lymph nodes (ALNs)^10–12^. Retrospective analysis from phase III SWOG S8814 trial demonstrated that 21-gene RS was prognostic in postmenopausal BC patients with HR-positive, HER2-negative and node-positive disease. Patients with low risk RS got little benefit from adjuvant chemotherapy, while high RS ones could receive more benefit from chemotherapy^10^. Data from ECOG E2197 showed that continuous RS was a highly significant independent predictor of recurrence for node-positive patients^11^. Similarly, the WGS Plan B trial also found an excellent survival outcome in node-positive, RS < 11 low risk patients treated with endocrine therapy alone, indicating a satisfactory prognostic value of RS^12,13^. Based on these findings, the 2018 NCCN Guidelines suggested to consider RS assay testing in selected patients with HR-positive, HER2-negative and pN1mi or pN1 disease, so as to guide adjuvant treatment choice^8^. Nevertheless, the impact of 21-gene RS results on adjuvant chemotherapy decision has not been fully understood in BC patients with positive ALN.

In the current study, we aim to evaluate whether 21-gene RS can influence adjuvant chemotherapy choice for patients with HR-positive, HER2-negative and pN1 BC, and to further analyze the adherence rate of adjuvant chemotherapy after 21-gene RS testing in clinical practice.

## Patients and Methods

### Study population

BC patients who met the following eligibility criteria were included in the study: (1) female gender; (2) invasive BC; (3) surgical procedure in Comprehensive Breast Health Center, Ruijin Hospital, Shanghai Jiao Tong University School of Medicine, Shanghai, China, between January 2014 and December 2018; (4) histologically proven positive involvement of 1 to 3 ALNs or ALN micro-metastases; (5) HR-positive, HER2-negative; (6) available 21-gene RS result. Exclusion criteria were as follows: (1) neoadjuvant therapy; (2) *de novo* stage IV BC. Patient information was retrospectively retrieved from Shanghai Jiao Tong University Breast Cancer Database (SJTU-BCDB).

### Histo-pathologic analysis

Tumor histo-pathologic analysis was performed in the Department of Pathology, Ruijin Hospital, Shanghai Jiao Tong University School of Medicine, Shanghai, China by experienced pathologists. The methods and criteria for immunohistochemistry (IHC) assessment of estrogen receptor (ER), progesterone receptor (PR), HER2 and Ki-67 were as described in our previous reports^14,15^. The cutoff of ER expression was set at 50% because the St. Gallen International Expert Consensus on the Primary Therapy of Early Breast Cancer 2009 has suggested that tumor cell staining for HR ≥ 50% indicated highly endocrine-responsive tumors^16^. HER2 negativity was identified according to the 2018 ASCO/CAP guidelines, which included IHC HER2 0, IHC HER2 1+, and IHC HER2 2+ with fluorescence *in situ* hybridization *HER2* non-amplified^17^. Molecular subtype classification was based on 2013 St. Gallen expert panel consensus, with Luminal A-like being defined as ER+/PR ≥ 20%/HER2-/Ki-67 < 14%, while Luminal B-like being defined as ER−/PR+/HER2- or ER+/HER2-/Ki-67 ≥ 14% or ER+/PR < 20%/HER2-^18^.

### Evaluation of 21-gene RS

The detailed information of 21-gene RS evaluation was described in our previous work^19^. RNA was extracted from three 10μm unstained sections of FFPE breast tumor tissue, which was prepared by experienced pathologists in the Department of Pathology, using RNeasy FFPE RNA kit (Qiagen, 73504, Germany). Reverse transcription was performed using Omniscript RT kit (Qiagen, 205111, Germany). Quantitative RT-PCR was accomplished using Premix Ex TaqTM (TaKaRa Bio, RR390A) in Applied Biosystems 7500 Real-Time PCR System (Foster City, CA). Gene expression was verified in triplicate, and normalized to five endogenous reference genes. Gene-specific normalized cycle threshold value was applied to calculate RS. For patients with multifocal disease, the highest RS was recorded.

### Treatment decision

Treatment choices pre- and post-RS were both decided through a two-round multidisciplinary team (MDT) meeting including surgical oncologists, medical oncologists, radiation oncologists, pathologists, BC specialized nurses, and other related specialists. After the completion of histo-pathologic analysis, a first-round MDT would be held to give an initial recommendation of adjuvant treatment regimen based on patients’ clinico-pathological features. For those who need additional information to guide treatment choice, MDT would recommend a 21-gene RS test. After receiving the RS result, a final treatment recommendation would be made through a second-round MDT based on traditional clinico-pathological features and RS. Frequently suggested chemotherapy regimen included EC-T, 4 cycles of epirubicin 90 mg/m^2^ and cyclophosphamide 600 mg/m^2^ every 21 days followed by 4 cycles of docetaxcel 100 mg/m^2^ every 21 days or 12 cycles of weekly paclitaxel 80 mg/m^2^; TC*4, 4 cycles of docetaxel 75 mg/m^2^ plus cyclophosphamide 600 mg/m^2^ every 21 days; TC*6, 6 cycles of docetaxel 75 mg/m^2^ plus cyclophosphamide 600 mg/m^2^ every 21 days. Actual chemotherapy usage and regimen were confirmed during follow-up, which was accomplished by the BC specialized nurses in our center.

### Statistical analysis

The 21-gene RS was calculated from the reference-normalized formula. Since the optimal RS cutoff in node-positive patients remains unknown, here we adopted two classifications. The classic classification divided patients into three risk groups: low RS (RS < 18), intermediate RS (18–30), and high RS (≥31), respectively. Another more specific classification was also presented (RS < 11, 11–17, 18–25, 26–30, and ≥31) based on classic and new TAILORx trial category classifications. Charlson Comorbidity Index was applied to evaluate patient comorbidity. Categorical variables were analyzed by using Chi-square test or Fisher’s exact test. Multivariate logistic regression was used to identify the impact factors for treatment recommendation. The change of treatment recommendation before and after 21-gene RS result was calculated by the subtraction between pre- and post-RS chemotherapy recommendation. Disease-free survival (DFS) was calculated from definitive surgery to the first proven local regional recurrence, distant metastasis, contralateral BC, second malignancy or death of any cause. Kaplan-Meier curve was applied to compare DFS between RS groups. Data were analyzed using IBM SPSS statistics software version 23 (SPSS, Inc., Chicago, IL). Two-sided *P* values < 0.05 were considered statistically significant.

### Ethical approval

This study was reviewed and approved by the independent Ethical Committees of Ruijin Hospital, Shanghai Jiao Tong University School of Medicine. All procedures performed in studies involving human participants were in accordance with the ethical standards of the institutional and/or national research committee and with the 1964 Helsinki declaration and its later amendments or comparable ethical standards. Informed consent was obtained from each patient.

## Results

### Baseline characteristics

**Figure 1:**
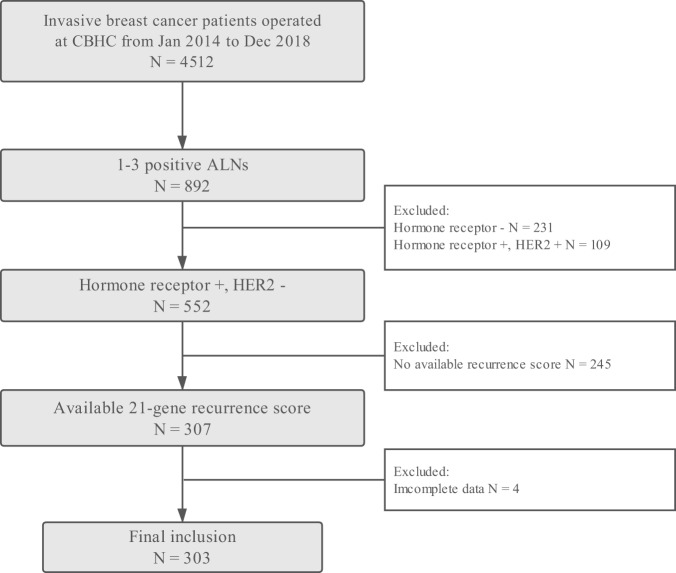
Flow chart of included patients. Abbreviations: ALN, axillary lymph node; CBHC, Comprehensive Breast Health Center; HER2, human epidermal growth factor receptor 2.

Overall, 303 women were enrolled in this study (Fig. 1). The baseline characteristics of the participants were presented in Table 1. The mean age was 59.41 ± 12.02 (range 30–89) years. Charlson Comorbidity Index was 0, 1, and ≥ 2 in 184, 81, and 38 patients, respectively. Invasive ductal carcinoma (IDC) was diagnosed in 279 out of 303 patients, while others had invasive lobular carcinoma, mucinous carcinoma, or mixed carcinoma. Grade I-II tumors were found in 73.27% (222/303) patients. There were 19.80%, 52.48%, 22.44%, and 5.28% patients with micro-metastases, one, two, and three positive ALN(s), respectively. All patients had ER-positive disease, of whom only 7 had ER staining in less than 50% BC cells. PR was less than 20% in 81 patients and 19 were PR-negative. One hundred and eighty-nine (62.38%) patients had Ki-67 ≥ 14%. Luminal A-like and Luminal B-like subtypes accounted for 26.07% and 73.39% of the study population.

**Table 1:** Baseline characteristics of study participants and impact factors for chemotherapy decision.

Characteristics	Total N = 303	Chemo^†^ N = 258 (%)	Non-Chemo^†^ N = 45 (%)	*P*-value
Age (years)				**<0.001**
<50	68 (22.44)	66 (25.58)	2 (4.44)	
50–70	188 (62.05)	173 (67.05)	15 (33.33)	
>70	47 (15.51)	19 (7.36)	28 (62.22)	
Menopausal status				**0.002**
Premenopausal	75 (24.75)	72 (27.91)	3 (6.67)	
Postmenopausal	228 (75.25)	186 (72.09)	42 (93.33)	
Comorbidity				**<0.001**
0	184 (60.73)	170 (65.89)	14 (31.11)	
1	81 (26.73)	67 (25.97)	14 (31.11)	
≥2	38 (12.54)	21 (8.14)	17 (37.78)	
Histologic type				0.145
IDC	279 (92.08)	240 (93.02)	39 (86.67)	
Non-IDC	24 (7.92)	18 (6.98)	6 (13.33)	
Tumor grade				**0.004**
I	17 (5.61)	11 (4.26)	6 (13.33)	
II	205 (67.66)	169 (65.50)	36 (80.00)	
III	81 (26.73)	78 (30.23)	3 (6.67)	
Tumor size (cm)				0.118
≤2	156 (51.49)	128 (49.61)	28 (62.22)	
>2	147 (48.51)	130 (50.39)	17 (37.78)	
Breast surgery				0.330
BCS	102 (33.66)	84 (32.56)	18 (40.00)	
Mastectomy	201 (66.34)	174 (67.44)	27 (60.00)	
Positive ALN(s)				0.333
Micro-metastasis	60 (19.80)	48 (18.60)	12 (26.67)	
1	159 (52.48)	134 (51.94)	25 (55.56)	
2	68 (22.44)	61 (23.64)	7 (15.56)	
3	16 (5.28)	15 (5.81)	1 (2.22)	
ER (%)				0.264
≥50	296 (97.69)	251 (97.29)	45 (100.00)	
<50	7 (2.31)	7 (2.71)	0 (0.00)	
PR (%)				**0.028**
≥20	222 (73.27)	183 (70.93)	39 (86.67)	
<20	81 (26.73)	75 (29.07)	6 (13.33)	
Ki-67 (%)				**<0.001**
<14	114 (37.62)	86 (33.33)	28 (62.22)	
≥14	189 (62.38)	172 (66.67)	17 (37.78)	
Molecular subtype				**<0.001**
Luminal A-like	79 (26.07)	57 (22.09)	22 (48.89)	
Luminal B-like	224 (73.93)	201 (77.91)	23 (51.11)	
21-gene RS				**<0.001**
Low RS	59 (19.47)	35 (13.57)	24 (53.33)	
Intermediate RS	178 (58.75)	158 (61.24)	20 (44.44)	
High RS	66 (21.78)	65 (25.19)	1 (2.22)	

^†^Chemo or Non-chemo was judged upon final multidisciplinary recommendation.

Abbreviations: ALN, axillary lymph node; BCS, breast conserving surgery; Chemo, chemotherapy; ER, estrogen receptor; IDC, invasive ductal carcinoma; PR, progesterone receptor; RS, recurrence score.

The number of pN1 patients receiving 21-gene RS was 66/763 (8.65%) before 2015, 53/176 (30.11%) in 2015, and 184/640 (28.75%) in 2016–2018. the application rate of 21-gene RS was 19.84% in pre-/perimenopausal patients, and 42.78% in postmenopausal patients. According to 21-gene RS, 59 (19.47%), 178 (58.75%), and 66 (21.78%) patients were categorized into low, intermediate, and high risk groups, respectively. Univariate (Supplementary Table S1) and multinomial logistic regression showed that the overall distribution of grade (*P* = 0.009), ER status (*P* = 0.009), and PR status (*P* < 0.001) had a significant difference among low, intermediate, and high risk groups (Supplementary Table S2). Those with RS ≥ 31 were less often of grade II tumors compared with intermediate risk patients (odds ratio [OR] 0.39, 95% confidence interval [CI] 0.20–0.75, *P* = 0.015). All 7 patients of ER < 50 were in the high risk group (*P* = 0.009). When compared to the patients with 21-gene RS 18–30, low RS patients were less often PR < 20% (OR 0.12, 95% CI 0.03–0.55, *P* = 0.007) while high risk ones were more possible PR < 20% (OR 2.57, 95% CI 1.27–5.21, *P* = 0.009).

### Impact factors for chemotherapy recommendation

**Figure 2:**
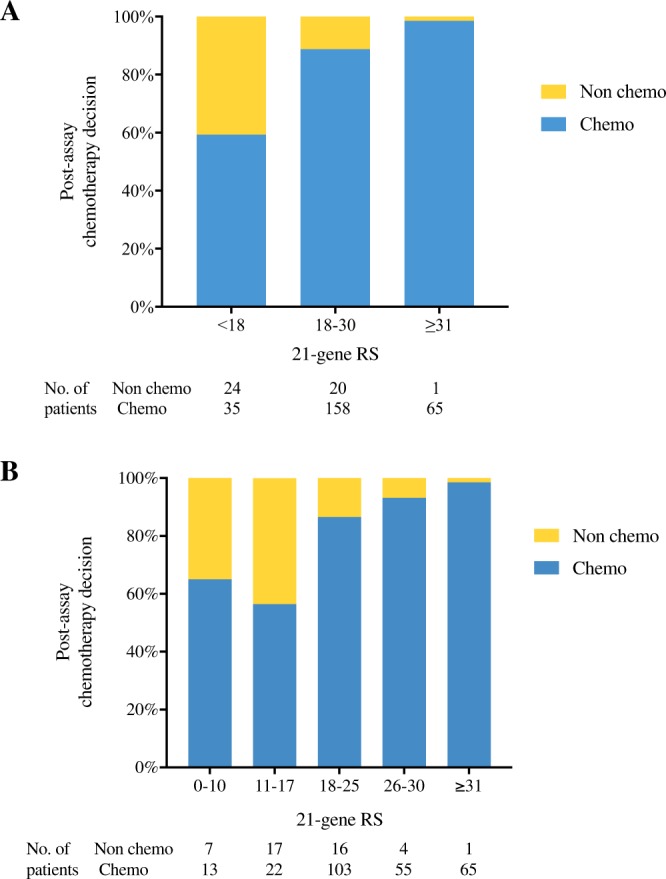
Distribution of post-assay chemotherapy recommendation by RS. Abbreviations: RS, recurrence score.

The distribution of post-assay chemotherapy recommendation by RS subgroups was shown in Fig. 2. Chemotherapy was recommended in 59.32%, 88.76% and 98.48% of low, intermediate and high risk patients, respectively (Fig. 2A). When further classified by RS, 65.00%, 56.41%, 86.55%, 93.22% and 98.48% patients with RS 0–10, 11–17, 18–25, 26–30 and ≥31 were recommended with chemotherapy (Fig. 2B).

Univariate and multivariate analysis revealed that age (*P* < 0.001), comorbidity (*P* = 0.013), and 21-gene RS (*P* < 0.001) were independent impact factors for chemotherapy recommendation in patients with pN1 BC (Table 2**)**. Compared with patients <50 years old, elder patients >70 years old were less likely to be recommended with chemotherapy (OR 0.01, 95% CI 0.00–0.11, *P* = 0.001). Those with comorbidity score of 2 or more had less possibility of chemotherapy recommendation compared to those without any comorbidity (OR 0.08, 95% CI 0.02–0.46, *P* = 0.004). Patients with intermediate RS (OR 6.58, 95% CI 2.37–18.31, *P* < 0.001) or high RS (OR 54.14, 95% CI 3.77–776.54, *P* = 0.003) were more likely to be recommended to receive chemotherapy than those with low RS.

**Table 2:** Multivariate analysis of impact factors for chemotherapy recommendation.

Characteristics	Odds ratio	95% Confidence interval	*P*-value
Age (years)			**<0.001**
<50	1.0		
50–70	0.20	0.01–3.47	0.271
>70	0.01	0.00–0.11	0.001
Menopausal status			0.653
Premenopausal	1.0		
Postmenopausal	1.79	0.14–22.72	
Comorbidity			**0.013**
0	1.0		
1	1.14	0.37–3.51	0.826
≥2	0.08	0.02–0.46	**0.004**
Tumor grade			0.230
I	1.0		
II	2.37	0.54–10.47	0.254
III	6.72	0.76–59.37	0.086
PR			0.869
<20	1.0		
≥20	1.15	0.23–5.66	
Ki-67 (%)			0.971
<14	1.0		
≥14	1.03	0.17–6.13	
Molecular subtype			0.156
Luminal A-like	1.0		
Luminal B-like	3.88	0.60–25.19	
21-gene RS			**<0.001**
Low RS	1.0		
Intermediate RS	6.58	2.37–18.31	**<0.001**
High RS	54.14	3.77–776.54	**0.003**

Abbreviations: RS, recurrence score.

When stratified by menopausal status, 3 out of 75 premenopausal patients omitted chemotherapy (Supplementary Table S3). For postmenopausal pN1 patients, elder age (*P* < 0.001), more comorbidities (*P* = 0.016), higher 21-gene RS (*P* = 0.001) were independent impact factors for chemotherapy recommendation (Table 3**)**. Compared to low RS patients, those with intermediate RS (OR 6.36, 95% CI 2.10–19.31, *P* = 0.001) or high RS (OR 48.89, 95% CI 3.38–708.44, *P* = 0.004) were more likely to receive chemotherapy recommendation.

**Table 3:** Impact factors for chemotherapy decision in postmenopausal patients (N = 228).

Characteristics	Univariate			Multivariate		
	Chemo^†^	Non-chemo^†^	*P*	OR	95% CI	*P*
Age (years)			<**0.001**			**<0.001**
<50	1 (0.54)	0 (0.00)		1.0		
50–70	166 (89.25)	14 (33.33)		0.00	0.00-	
>70	19 (10.22)	28 (66.67)		0.00	0.00-	
Comorbidity			**0.001**			**0.016**
0	133 (71.51)	20 (47.62)		1.0		
1	44 (23.66)	14 (33.33)		1.12	0.37–3.44	0.840
≥2	9 (4.84)	8 (19.05)		0.09	0.02–0.50	**0.006**
Tumor grade			**0.002**			0.439
I	7 (3.76)	4 (9.52)		1.0		
II	118 (63.44)	35 (83.33)		1.09	0.18–6.61	0.925
III	61 (32.80)	3 (7.14)		3.11	0.28–34.69	0.357
PR (%)			**0.015**			0.845
≥20	62 (33.33)	6 (14.29)		1.0		
<20	124 (66.67)	36 (85.71)		1.17	0.24–5.72	
Ki-67 (%)			**0.001**			0.977
<14	60 (32.26)	25 (59.52)		1.0		
≥14	126 (67.74)	17 (40.48)		1.03	0.17–6.14	
Molecular subtype			**<0.001**			0.179
Luminal A-like	34 (18.28)	19 (45.24)		1.0		
Luminal B-like	152 (81.72)	23 (54.76)		3.67	0.55–24.44	
21-gene RS			**<0.001**			**0.001**
Low RS	26 (13.98)	22 (52.38)		1.0		
Intermediate RS	110 (59.14)	19 (45.24)		6.36	2.10–19.31	**0.001**
High RS	50 (26.88)	1 (2.38)		48.89	3.38–708.44	**0.004**

^†^Chemo or Non-chemo was judged upon final multidisciplinary recommendation.

Abbreviations: Chemo, chemotherapy; CI, confidence interval; OR, odds ratio; PR, progesterone receptor; RS, recurrence score.

### Change in chemotherapy recommendation before and after 21-gene RS assay

**Figure 3:**
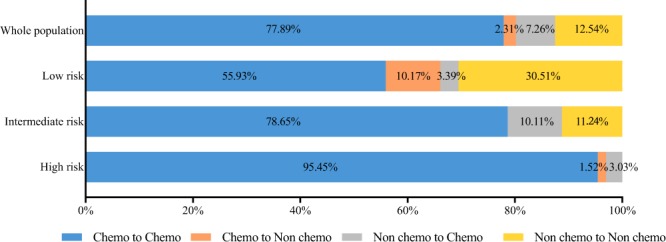
Distribution of chemotherapy recommendation before and after 21-gene RS testing. Abbreviations: Chemo, chemotheray; Non chemo, non chemotherapy; RS, recurrence score.

The distribution of pre- and post-RS chemotherapy recommendation was presented in Fig. 3. Overall, physician’s treatment recommendation changed for 9.57% (29/303; Table 4) patients. The most apparent alteration was found in the low RS group, with 6 (10.17%) patients changing from chemotherapy to no chemotherapy, and 2 (3.39%) patients reversely. Eighteen (10.11%) intermediate RS patients switched to receive chemotherapy, whose median was 24.5 (18.0–29.0). Two patients in the high risk group were changed to receive chemotherapy, while another patient was recommended to omit chemotherapy after MDT, since this patient was 82 years’ old with medical history of hypertension, type 2 diabetes, and severe cerebral infarction. Supplementary Table S5 compared the clinico-pathological features between patients with or without chemotherapy recommendation alteration. Tumor grade, tumor size, positive lymph node number, Ki-67 level and molecular subtype were significantly associated with treatment recommendation change in univariate model. Multivariate analysis showed that patients with greater tumor size (>2 cm *vs* ≤2 cm, OR 0.36, 95% CI 0.15–0.89, *P* = 0.026; Supplementary Table S6) or 2 positive lymph nodes (2 *vs* micro-metastasis, OR 0.07, 95% CI 0.01–0.58, *P* = 0.014) were less likely undergo treatment recommendation change.

**Table 4:** Chemotherapy recommendation before, after 21-gene RS assay and actual use.

Post-RS	Pre-RS		Pre-RS to Post-RS change (%)	Actual application		Actual use adherence to post-RS testing (%)
	Chemo	Non-chemo		Chemo	Non-chemo	
Whole population						
Chemo	236	22	29/303 (9.57%)	245	13	287/303
Non-chemo	7	38		3	42	(94.72%)
Low RS (RS < 18)						
Chemo	33	2	8/59 (13.56%)	35	0	58/59
Non-chemo	6	18		1	23	(98.31%)
Intermediate RS (RS 18–30)						
Chemo	140	18	18/178 (10.11%)	147	11	165/178
Non-chemo	0	20		2	18	(92.70%)
High RS (RS ≥ 31)						
Chemo	63	2	3/66	63	2	64/66
Non-chemo	1	0	(4.55%)	0	1	(96.97%)

Abbreviations: Chemo, chemotherapy; RS, recurrence score.

Table 5 showed the chemotherapy regimen recommended before and after 21-gene RS result. In the low RS group, TC*4 was the most frequently recommended regimen both before (47.46%) and after (40.68%) RS assay testing. For patients with intermediate risk, EC-T was suggested in 33.15% and 33.15% patients pre- and post-assay, respectively. Recommendation of TC*4 increased from 36.52% to 47.19% after receiving RS result. Moreover, in the high risk group, EC-T was proposed to 57.58% before and 63.64% after having 21-gene RS results.

**Table 5:** Chemotherapy regimen recommendation before and after 21-gene RS assay.

21-gene RS	Regimen	Recommendation before 21-gene RS, N (%)	Recommendation after 21-gene RS, N (%)	Therapy actually achieved, N (%)
Whole population	EC-T	104 (34.32)	104 (34.32)	93 (30.69)
	TC*4	113 (37.29)	122 (40.26)	118 (38.94)
	TC*6	24 (7.92)	27 (8.91)	25 (8.25)
	Other	2 (0.66)	5 (1.65)	12 (3.96)
	None	60 (19.80)	45 (14.85)	55 (18.15)
RS < 18 N = 59	EC-T	7 (11.86)	3 (5.08)	2 (3.39)
	TC*4	28 (47.46)	24 (40.68)	26 (44.07)
	TC*6	3 (5.08)	6 (10.17)	6 (10.17)
	Other	1 (1.69)	2 (3.39)	2 (3.39)
	None	20 (33.90)	24 (40.68)	23 (38.98)
RS 18–30 N = 178	EC-T	59 (33.15)	59 (33.15)	55 (30.90)
	TC*4	65 (36.52)	84 (47.19)	79 (44.38)
	TC*6	16 (8.99)	13 (7.30)	10 (5.62)
	Other	0 (0.00)	2 (1.12)	5 (2.81)
	None	38 (21.35)	20 (11.24)	29 (16.29)
RS ≥ 31 N = 66	EC-T	38 (57.58)	42 (63.64)	36 (54.55)
	TC*4	20 (30.30)	14 (21.21)	13 (19.70)
	TC*6	5 (7.58)	8 (12.12)	9 (13.64)
	Other	1 (1.52)	1 (1.52)	5 (7.58)
	None	2 (3.03)	1 (1.52)	3 (4.55)

Abbreviations: EC-T, 4 cycles of epirubicin and cyclophosphamide every 21 days followed by 4 cycles of docetaxcel every 21 days or 12 cycles of weekly paclitaxel; RS, recurrence score; TC*4, 4 cycles of docetaxel plus cyclophosphamide every 21 days; TC*6, 6 cycles of docetaxel plus cyclophosphamide every 21 days.

### Actual adjuvant chemotherapy usage and disease outcomes

With respect to the actual adjuvant chemotherapy usage, 16 patients didn’t follow the treatment recommendation (Table 4), with adherence rate with MDT decision 94.72% (287/303) in the whole population. Regarding patients with different RS, the adherence rate with MDT decision about adjuvant chemotherapy was 98.31% (58/59), 92.70% (165/178), and 96.97% (64/66) in the low RS, intermediate RS, and high RS groups, respectively (*P* = 0.162)

After a median follow-up of 21.17 (range 1.38 to 55.43) months, 11 (3.63%) DFS events were observed, including 4 local regional recurrences, 5 distant metastases, 1 second primary malignancy, and 1 death. The detailed information of the 11 patients with DFS events were summarized in Supplementary Table S4. There was no significant DFS difference among different risk groups (*P* = 0.225).

## Discussion

In this study, we included 303 HR+/HER2- and pN1 BC patients with 21-gene RS records. The distribution of RS was 19.47%, 58.75%, and 21.78% for low, intermediate, and high risk group, respectively. Chemotherapy was recommended for 258 patients after MDT meeting. We found that age, comorbidity, and 21-gene RS were independently associated with chemotherapy recommendation in the whole population and for postmenopausal patients. Treatment recommendation changed for 9.57% patients after RS results. The overall adherence rate of actual chemotherapy usage to MDT decision was 94.72% (287/303). To our knowledge, this is the first and largest study in Chinese population to focus on 21-gene RS and adjuvant chemotherapy decision in ALN + BC patients.

Earlier data from our center revealed that among node-negative and node-positive BC population receiving 21-gene RS tests, 26.1%, 49.3% and 24.6% were categorized into low, intermediate and high RS groups, respectively^19^. Tumor grade, PR status, and Ki-67 were significantly associated with RS category in the whole population^19^, which was consistent with evidence from other centers^20,21^. In this current study, grade, ER status, and PR status were identified as independent factors associated with RS in pN1 patients.

We found that the RS category was independently associated with chemotherapy recommendation in pN1 patients in our study, which was in consistency with previous findings^22–24^. For low RS pN1 patients, 40.68% (24/59) were exempt from chemotherapy. Based on Surveillance, Epidemiology, and End Results (SEER) database, Roberts *et al*. concluded that the 5-year breast cancer specific survival (BCSS) was 98.9%, 99.4%, 97.1% and, 95.1% for those RS < 18 with micro-metastases, 1, 2 and, 3 positive ALN(s), respectively^25^. Similarly, in the WGS Plan B trial, the 5-year DFS in RS < 11 N + patients treated with endocrine therapy alone was 94% after a median follow-up of 55 months^12,13^. Nevertheless, given the relatively short follow-up for ER-positive disease, and an increase in DFS events of 5.6% at 5-year compared with the 3-year results, we cannot spare all RS < 11 N + patients from chemotherapy. The optimal cutoff in node-positive patients remains unclear. Actually, the two classification (classic classification of 18–30, the new TAILORx classification of 11–25) are both in use in clinical practice. Based on previous evidence, the 18–30 is more frequently adopted when doing prognostic analysis (for example in transATAC trial), while the 11–25 is more frequently applied when studying the predictive value. We carried out a Chi-square test to compare two different classifications, and the Chi-square value is 22.6 when applying the new classification, compared to 42.2 when applying the classic classification. In addition, we found that the chemotherapy recommendation rates were similar between patients with RS < 11, and RS 11–17 (65.00% *vs* 56.41%, *P* = 0.525, Fig. 2B), but much lower than those with RS ≥ 18 (*P* < 0.001). As a result, we adopted the cutoff of 18–30 in the current study. The ongoing prospective randomized phase III RxPONDER trial is designed to study the efficacy of adjuvant endocrine therapy with or without chemotherapy in HR-positive, HER2-negative patients with 1–3 positive ALNs, RS ≤ 25. The results are awaited to evaluate the interaction of RS and chemotherapy benefit in pN1 patients, and to estimate a clinical meaningful cutoff point for chemotherapy recommendation in this subgroup^26^.

The influence of 21-gene RS on chemotherapy usage has been noticed. According to data from other centers, the change in adjuvant treatment recommendations for node-positive patients after 21-gene RS assay was 21%-39%, in the direction of exempting from chemotherapy^23,27–30^. Meanwhile, our data showed that only 9.57% patients received inconsistent adjuvant treatment recommendation before and after 21-gene RS assay, and more patients of intermediate risk group were recommended with chemotherapy after RS results. One possible reason was that, in previous studies of other centers, chemotherapy recommendation both pre- and post-21-gene RS assay were made by individual clinician or only one-round MDT, which might be more frequently influenced by RS results. In our clinical practice, however, chemotherapy recommendation pre- and post-21-gene RS assay were both made through two-round MDT meetings. Our previous study about MDT found that MDT changed the traditional single-disciplinary treatment mode, and showed significant advantages in providing better treatment options for patients^31^. Such effect of MDT decision might thus dilute the impact of 21-gene RS on chemotherapy recommendation.

Previous studies have indicated that 21-gene RS assay provides additional prognostic information beyond clinico-pathological features. For example, Wang *et al*. analyzed data of 4059 T_1–2_N_1_M_0_ patients with ER-positive, HER2-negative diseases with available 21-gene RS results from the SEER database. Their study indicated that the RS risk categories were positively associated with pathological prognostic stages (*P* < 0.001) based on the 8^th^ edition of American Joint Commission on Cancer (AJCC). The RS risk category was an independent prognostic factor for BCSS and overall survival^32^. Similarly, another SEER database-based study found that RS result was a strong predictor of BCSS for patients with micro-metastases or 1–3 positive ALNs (*P* < 0.001)^29^. In addition, other retrospective studies demonstrated that RS result was an independent impact factor for local regional recurrence (Hazard ratio = 2.59, 95% CI 1.28–5.26, *P* = 0.008)^33^ and distant recurrence (Hazard ratio = 3.47, 95% CI 1.64–7.38, *P* = 0.002)^25^ in ER-positive, node-positive patients. Our study found that after a median follow-up of 21.17 months, 11 (3.63%) DFS events were observed in these node-positive patients. Due to the relative short period of follow-up and small number of DFS events, we cannot assess the prognostic value of 21-gene RS in ALN positive BC patients, which warranted further evaluation.

Several limitations existed in the current study. To begin with, 245 (44.38%) pN1 patients didn’t receive a 21-gene RS test since not enough data supported the application of 21-gene RS in node-positive patients at time of diagnosis, which might introduce bias. Secondly, the RS distribution differed from previous data from other clinical trials like SWOG S8814 and transATAC. This might be explained by the difference in clinico-pathological features between the enrolled cohorts. For example, in the SWOG8814 trial, 35.7%, 52.9% and 11.4% patients had grade 1,2 and 3 tumor, compared to 5.6%, 67.7% and 26.7% in our cohort. Moreover, given that there was limited evidence about the application of 21-gene RS in node-positive patients, we recommended 21-gene RS testing for N + patients increasingly but not routinely, only after the publication of SWOG S8814 and WGS Plan B trials, thus the follow-up was too short to clarify the prognostic effect of 21-gene RS, which needed continuous follow-up.

## Conclusions

In conclusion, 21-gene RS category independently influenced chemotherapy recommendation in HR+/HER2- BC patients with 1–3 positive ALNs. Chemotherapy recommendation changed for 9.57% patients after 21-gene RS results, which provide relatively little information to guide adjuvant treatment decision in these patients before the publication of RxPONDER trial. After 21-gene RS testing, ALN positive patients had a good adherence to MDT decision. Further analysis is warranted to clarify the prognostic and chemotherapy predictive value of 21-gene RS in pN1 breast cancer patients.

## Data Availability

The datasets analysed during the current study are available from the corresponding authors on reasonable request.

## Supplementary information

Supplementary Tables
