# Supplementary material for: 21-Gene Recurrence Score and Adjuvant Chemotherapy Decision for Breast Cancer Patients with Positive Lymph Nodes
Source: Sci Rep. 2019 Sep 11;9:13123. doi: 10.1038/s41598-019-49644-6 (PMC6739381; doi:10.1038/s41598-019-49644-6)
Supplement: Supplementary file 1 — Supplementary Tables [file 41598_2019_49644_MOESM1_ESM.docx]

**Supplementary Information**

**21-Gene Recurrence Score and Adjuvant Chemotherapy Decision for Breast Cancer Patients with Positive Lymph Nodes**

Yiwei Tong^1^, Jiayi Wu^1^, Ou Huang^1^, Jianrong He^1^, Li Zhu^1^, Weiguo Chen^1^, Yafen Li^1^, Xiaosong Chen^1^*, Kunwei Shen^1^*

**Supplementary Table S1. Baseline characteristics of study participants (N = 303)**

| **Characteristics** | **Total** | | **Low risk** | | **Intermediate risk** | | **High risk** | ***P*-value** |
| --- | --- | --- | --- | --- | --- | --- | --- | --- |
|  | **N=303** | | **N=59 (%)** | | **N=178 (%)** | | **N=66 (%)** |  |
| Age (years) |  | |  | |  | |  | 0.111 |
| $<$50 | 68 (22.44) | | 10 (16.95) | | 45 (25.28) | | 13 (19.70) |  |
| 50-70 | 188 (62.05) | | 34 (57.63) | | 108 (60.67) | | 46 (69.70) |  |
| $>$70 | 47 (15.51) | | 15 (25.42) | | 25 (14.04) | | 7 (10.61) |  |
| Menopausal status | |  | |  | |  | | 0.356 |
| Premenopausal | 75 (24.75) | | 11 (18.64) | | 49 (27.53) | | 15 (22.73) |  |
| Postmenopausal | 228 (75.25) | | 48 (81.36) | | 129 (72.47) | | 51 (77.27) |  |
| Comorbidity |  | |  | |  | |  | 0.504 |
| 0 | 222 (73.27) | | 41 (69.49) | | 131 (73.60) | | 50 (75.76) |  |
| 1 | 63 (20.79) | | 14 (23.73) | | 39 (21.91) | | 10 (15.15) |  |
| ≥2 | 18 (5.94) | | 4 (6.78) | | 8 (4.49) | | 6 (9.09) |  |
| Histologic type |  | |  | |  | |  | 0.318 |
| IDC | 279 (92.08) | | 52 (88.14) | | 164 (92.13) | | 63 (95.45) |  |
| Non-IDC | 24 (7.92) | | 7 (11.86) | | 14 (7.87) | | 3 (4.55) |  |
| Tumor grade |  | |  | |  | |  | **<0.001** |
| I | 17 (5.61) | | 7 (11.86) | | 10 (5.62) | | 0 (0.00) |  |
| II | 205 (67.66) | | 44 (74.58) | | 129 (72.47) | | 32 (48.48) |  |
| III | 81 (26.73) | | 8 (13.56) | | 39 (21.91) | | 34 (51.52) |  |
| Tumor size (cm) |  | |  | |  | |  | 0.497 |
| $\leq$2 | 156 (51.49) | | 30 (50.85) | | 96 (53.93) | | 30 (45.45) |  |
| $>$2 | 147 (48.51) | | 29 (49.15) | | 82 (46.07) | | 36 (54.55) |  |
| Breast surgery |  | |  | |  | |  | 0.282 |
| BCS | 102 (33.66) | | 21 (35.59) | | 54 (30.34) | | 27 (40.91) |  |
| Mastectomy | 201 (66.34) | | 38 (64.41) | | 124 (69.66) | | 39 (59.09) |  |
| Positive ALN | | | | | | | | 0.217 |
| Micro-metastasis | 60 (19.80) | | 7 (11.86) | | 41 (23.03) | | 12 (18.18) |  |
| 1 | 159 (52.48) | | 35 (59.32) | | 83 (46.63) | | 41 (62.12) |  |
| 2 | 68 (22.44) | | 13 (22.03) | | 45 (25.28) | | 10 (15.15) |  |
| 3 | 16 (5.28) | | 4 (6.78) | | 9 (5.06) | | 3 (4.55) |  |
| ER (%) |  | |  | |  | |  | **<0.001** |
| $\geq$50 | 296 (97.69) | | 59 (100.00) | | 178 (100.00) | | 59 (89.39) |  |
| $<$50 | 7 (2.31) | | 0 (0.00) | | 0 (0.00) | | 7 (10.61) |  |
| PR (%) |  | |  | |  | |  | **<0.001** |
| $\geq$20 | 222 (73.27) | | 57 (96.61) | | 135 (75.84) | | 30 (45.45) |  |
| $<$20 | 81 (26.73) | | 2 (3.39) | | 43 (24.16) | | 36 (54.55) |  |
| Ki-67 (%) |  | |  | |  | |  | **<0.001** |
| $<$14 | 114 (37.62) | | 29 (49.15) | | 75 (42.13) | | 10 (15.15) |  |
| $\geq$14 | 189 (62.38) | | 30 (50.85) | | 103 (57.87) | | 56 (84.85) |  |
| Molecular subtype |  | |  | |  | |  | **<0.001** |
| Luminal A-like | 79 (26.07) | | 25 (42.37) | | 51 (28.65) | | 3 (4.55) |  |
| Luminal B-like | 224 (73.93) | | 34 (57.63) | | 127 (71.35) | | 63 (95.45) |  |

Abbreviations: RS, recurrence score; IDC, invasive ductal carcinoma; BCS, breast conserving surgery; ALN, axillary lymph node; ER, estrogen receptor; PR, progesterone receptor.

**Supplementary Table S2. Baseline characteristics stratified by 21-gene RS** ^†^

|  | Intermediate risk (N=178) | | |  | High risk (N=66) | | |  | *P*-value |
| --- | --- | --- | --- | --- | --- | --- | --- | --- | --- |
|  | **OR** | **95% CI** | ***P*-value** |  | **OR** | **95% CI** | ***P*-value** |  |  |
| Tumor grade |  |  |  |  |  |  |  |  | **0.009** |
| I | 0.37 | 0.09-1.51 | 0.374 |  | 0.00 | 0.00- | 0.997 |  |  |
| II | 0.64 | 0.26-1.55 | 0.324 |  | 0.25 | 0.09-0.67 | **0.006** |  |  |
| III | 1.0 |  |  |  | 1.0 |  |  |  |  |
| ER (%) |  |  |  |  |  |  |  |  | **0.009** |
| <50 | 0.13 | 0.13-0.13 |  |  | $\infty$ | 0-$\infty$ | 0.998 |  |  |
| ≥50 | 1.0 |  |  |  | 1.0 |  |  |  |  |
| PR (%) |  |  |  |  |  |  |  |  | **<0.001** |
| <20 | 8.40 | 1.81-38.98 | **0.007** |  | 21.59 | 4.32-107.93 | **<0.001** |  |  |
| ≥20 | 1.0 |  |  |  | 1.0 |  |  |  |  |
| Ki-67 (%) |  |  |  |  |  |  |  |  | 0.500 |
| <14 | 1.00 | 0.29-3.48 | 1.000 |  | 0.56 | 0.12-2.51 | 0.447 |  |  |
| ≥14 | 1.0 |  |  |  | 1.0 |  |  |  |  |
| Luminal subtype |  |  |  |  |  |  |  |  | 0.643 |
| Luminal A-like | 0.94 | 0.27-3.29 | 0.925 |  | 0.47 | 0.07-3.04 | 0.424 |  |  |
| Luminal B-like | 1.0 |  |  |  | 1.0 |  |  |  |  |

^†^ The reference category for subtype characteristics is intermediate risk group (RS 18-30).

Abbreviations: RS, recurrence score; OR, odds ratio; CI, confidence interval; ER, estrogen receptor; PR, progesterone receptor.

**Supplementary Table S3. Impact factors for chemotherapy decision in premenopausal patients (N = 75)**

| **Characteristics** | **Total** | **Chemo**^†^  **N=72 (%)** | **Non-Chemo**^†^  **N=3 (%)** |
| --- | --- | --- | --- |
|  | **N=75** |  |  |
| Age (years) |  |  |  |
| $<$50 | 67 (89.33) | 65 (90.28) | 2 (66.67) |
| 50-70 | 8 (10.67) | 7 (9.72) | 1 (33.33) |
| Comorbidity |  |  |  |
| 0 | 69 (92.00) | 66 (91.67) | 3 (100.00) |
| 1 | 5 (6.67) | 5 (6.94) | 0 (0.00) |
| ≥2 | 1 (1.33) | 1 (1.39) | 0 (0.00) |
| Histologic type |  |  |  |
| IDC | 70 (93.33) | 67 (93.06) | 3 (100.00) |
| Non-IDC | 5 (6.67) | 5 (6.94) | 0 (0.00) |
| Tumor grade |  |  |  |
| I | 6 (8.00) | 4 (5.56) | 2 (66.67) |
| II | 52 (69.33) | 51 (70.83) | 1 (33.33) |
| III | 17 (22.67) | 17 (23.61) | 0 (0.00) |
| Tumor size (cm) |  |  |  |
| $\leq$2 | 43 (57.33) | 41 (56.94) | 2 (66.67) |
| $>$2 | 32 (42.67) | 31 (43.06) | 1 (33.33) |
| Breast surgery |  |  |  |
| BCS | 29 (38.67) | 28 (38.89) | 1 (33.33) |
| Mastectomy | 46 (61.33) | 44 (61.11) | 2 (66.67) |
| Positive ALN(s) |  |  |  |
| Micro-metastasis | 31 (41.33) | 29 (40.28) | 2 (66.67) |
| 1 | 35 (46.67) | 34 (47.22) | 1 (33.33) |
| 2 | 9 (12.00) | 9 (12.50) | 0 (0.00) |
| ER (%) |  |  |  |
| $\geq$50 | 1 (1.33) | 1 (1.39) | 0 (0.00 |
| $<$50 | 74 (98.67) | 71 (98.61) | 3 (100.00) |
| PR (%) |  |  |  |
| $\geq$20 | 13 (17.33) | 13 (18.06) | 0 (0.00) |
| $<$20 | 62 (82.67) | 59 (81.94) | 3 (100.00) |
| Ki-67 (%) |  |  |  |
| $<$14 | 29 (38.67) | 26 (36.11) | 3 (100.00) |
| $\geq$14 | 46 (61.33) | 46 (63.89) | 0 (0.00) |
| Molecular subtype |  |  |  |
| Luminal A-like | 26 (34.66) | 23 (31.94) | 3 (100.00) |
| Luminal B-like | 49 (65.34) | 49 (68.06) | 0 (0.00) |
| 21-gene RS |  |  |  |
| Low RS | 11 (14.67) | 9 (12.50) | 2 (66.67) |
| Intermediate RS | 49 (65.33) | 48 (66.67) | 1 (33.33) |
| High RS | 15 (20.00) | 15 (20.83) | 0 (0.00) |

^†^ Chemo or Non-chemo was judged upon final multidisciplinary recommendation.

Abbreviations: ALN, axillary lymph node; BCS, breast conserving surgery; Chemo, chemotherapy; ER, estrogen receptor; IDC, invasive ductal carcinoma; PR, progesterone receptor; RS, recurrence score.

**Supplementary Table S4. Detailed information of patients with disease-free events**

| No. | RS | Event | Age | Histology | pTN | Molecular subtype | Post-assay chemo recommendation | Actual chemo usage |
| --- | --- | --- | --- | --- | --- | --- | --- | --- |
| 1 | 17 | Bone and lung M | 36 | IDC grade II | pT2N1 | Luminal B-like | TC*4 | TC*4 |
| 2 | 22 | Occipital skin M | 67 | IDC grade II | pT1N1 | Luminal B-like | TC*4 | Patient refusal |
| 3 | 23 | LRR (LNR) | 59 | IDC grade II | pT1N1 | Luminal B-like | TC*4 | Patient refusal |
| 4 | 24 | Liver M | 65 | IDC grade II | pT2N1 | Luminal B-like | EC-T | EC-T |
| 5 | 25 | Thyroid papillary carcinoma | 56 | IDC grade III | pT1N1 | Luminal B-like | TC*4 | TC*4 |
| 6 | 27 | LRR (IBTR) | 68 | IDC grade III | pT1N1 | Luminal B-like | TC*6 | Patient refusal |
| 7 | 32 | LRR (LNR) | 70 | IDC grade III | pT1N1 | Luminal B-like | EC-T | Patient refusal |
| 8 | 36 | LRR (LNR) | 76 | IDC grade III | pT2N1 | Luminal B-like | TC*4 | TC*4 |
| 9 | 38 | Bone M | 60 | IDC grade II | pT1N1 | Luminal B-like | TC*4 | TC*4 |
| 10 | 41 | Liver M | 53 | IDC grade III | pT2N1 | Luminal B-like | EC-T | EC-T |
| 11 | 50 | BC-related death | 67 | IDC grade III | pT1N1 | Luminal B-like | EC-T | AC-wP |

Abbreviations: RS, recurrence score; chemo, chemotherapy; M, metastasis; IDC, invasive ductal carcinoma; TC*4, 4 cycles of docetaxel plus cyclophosphamide every 21 days; EC-T, 4 cycles of epirubicin and cyclophosphamide every 21 days followed by 4 cycles of docetaxcel every 21 days; LRR, local regional recurrence; IBTR, ipsilateral breast tumor recurrence; TC*6, 6 cycles of docetaxel plus cyclophosphamide every 21 days; LNR, regional lymph nodes recurrence; BC, breast cancer; AC-wP, 4 cycles of doxorubicin and cyclophosphamide every 21 days followed by 12 weeks of weekly paclitaxel.

**Supplementary Table S5. Clinico-pathological characteristics of study participants with or without chemotherapy decision alteration**

| **Characteristics** | **Total** | **No change**  **N=274 (%)** | **Change**  **N=29 (%)** | ***P*-value** |
| --- | --- | --- | --- | --- |
|  | **N=303** |  |  |  |
| Age (years) |  |  |  | 0.125 |
| $<$50 | 68 (22.44) | 58 (21.17) | 10 (34.48) |  |
| 50-70 | 188 (62.05) | 175 (63.87) | 13 (44.83) |  |
| $>$70 | 47 (15.51) | 41 (14.96) | 6 (20.69) |  |
| Menopausal status | |  |  | 0.202 |
| Premenopausal | 75 (24.75) | 65 (23.72) | 10 (34.48) |  |
| Postmenopausal | 228 (75.25) | 209 (76.28) | 19 (65.52) |  |
| Comorbidity |  |  |  | 0.315 |
| 0 | 184 (60.73) | 166 (60.58) | 18 (62.07) |  |
| 1 | 81 (26.73) | 73 (26.64) | 8 (27.59) |  |
| ≥2 | 38 (12.54) | 35 (12.77) | 3 (10.34) |  |
| Histologic type |  |  |  | 0.611 |
| IDC | 279 (92.08) | 253 (92.34) | 26 (89.66) |  |
| Non-IDC | 24 (7.92) | 21 (7.66) | 3 (10.34) |  |
| Tumor grade |  |  |  | **0.009** |
| I | 17 (5.61) | 14 (5.11) | 3 (10.34) |  |
| II | 205 (67.66) | 180 (65.69) | 25 (86.21) |  |
| III | 81 (26.73) | 80 (29.20) | 1 (3.45) |  |
| Tumor size (cm) |  |  |  | **0.018** |
| $\leq$2 | 156 (51.49) | 135 (49.27) | 21 (72.41) |  |
| $>$2 | 147 (48.51) | 139 (50.73) | 8 (27.59) |  |
| Breast surgery |  |  |  | 0.922 |
| BCS | 102 (33.66) | 92 (33.58) | 10 (34.48) |  |
| Mastectomy | 201 (66.34) | 182 (66.42) | 19 (65.52) |  |
| Positive ALN(s) |  |  |  | **0.019** |
| Micro-metastasis | 60 (19.80) | 51 (18.61) | 9 (31.03) |  |
| 1 | 159 (52.48) | 140 (51.09) | 19 (65.52) |  |
| 2 | 68 (22.44) | 67 (24.45) | 1 (3.45) |  |
| 3 | 16 (5.28) | 16 (5.84) | 0 (0.00) |  |
| ER (%) |  |  |  | 0.384 |
| $\geq$50 | 296 (97.69) | 267 (97.45) | 29 (100.00) |  |
| $<$50 | 7 (2.31) | 7 (2.55) | 0 (0.00) |  |
| PR (%) |  |  |  | 0.225 |
| $\geq$20 | 222 (73.27) | 198 (72.26) | 24 (82.76) |  |
| $<$20 | 81 (26.73) | 76 (27.74) | 5 (17.24) |  |
| Ki-67 (%) |  |  |  | **0.004** |
| $<$14 | 114 (37.62) | 96 (35.04) | 18 (62.07) |  |
| $\geq$14 | 189 (62.38) | 178 (64.96) | 11 (37.93) |  |
| Molecular subtype |  |  |  | **0.016** |
| Luminal A-like | 79 (26.07) | 66 (24.09) | 13 (44.83) |  |
| Luminal B-like | 224 (73.93) | 208 (75.91) | 16 (55.17) |  |

Abbreviations: ALN, axillary lymph node; BCS, breast conserving surgery; ER, estrogen receptor; IDC, invasive ductal carcinoma; PR, progesterone receptor; RS, recurrence score.

**Supplementary Table S6. Multivariate analysis of impact factors for chemotherapy recommendation alteration**

| **Characteristics** | **Odds ratio** | **95% Confidence interval** | ***P*-value** |
| --- | --- | --- | --- |
| Tumor grade |  |  | 0.152 |
| I | 1.0 |  |  |
| II | 1.16 | 0.28-4.88 | 0.842 |
| III | 0.15 | 0.01-1.85 | 0.139 |
| Tumor size (cm) |  |  | **0.026** |
| $\leq$2 | 1.0 |  |  |
| $>$2 | 0.36 | 0.15-0.89 |  |
| Positive ALN(s) |  |  | 0.102 |
| Micro-metastasis | 1.0 |  |  |
| 1 | 0.86 | 0.35-2.13 | 0.745 |
| 2 | 0.07 | 0.01-0.58 | **0.014** |
| 3 | 0.00 | 0.00- | 0.998 |
| Ki-67 (%) |  |  | 0.167 |
| $<$14 | 1.0 |  |  |
| $\geq$14 | 0.42 | 0.13-1.43 |  |
| Molecular subtype |  |  | 0.971 |
| Luminal A-like | 1.0 |  |  |
| Luminal B-like | 0.98 | 0.30-3.22 |  |

Abbreviations: ALN, axillary lymph node.
